# Neuroinflammation Involved in Diabetes-Related Pain and Itch

**DOI:** 10.3389/fphar.2022.921612

**Published:** 2022-06-20

**Authors:** Xiao-Xia Fang, Heng Wang, Hao-Lin Song, Juan Wang, Zhi-Jun Zhang

**Affiliations:** ^1^ Department of Human Anatomy, School of Medicine, Nantong University, Nantong, China; ^2^ Department of Medical Functional Laboratory, School of Medicine, Nantong University, Nantong, China

**Keywords:** neuroinflammation, diabetes mellitus, diabetic pain, diabetic itch, sensitization

## Abstract

Diabetes mellitus (DM) is a global epidemic with increasing incidence, which results in diverse complications, seriously affects the patient quality of life, and brings huge economic burdens to society. Diabetic neuropathy is the most common chronic complication of DM, resulting in neuropathic pain and chronic itch. The precise mechanisms of diabetic neuropathy have not been fully clarified, hindering the exploration of novel therapies for diabetic neuropathy and its terrible symptoms such as diabetic pain and itch. Accumulating evidence suggests that neuroinflammation plays a critical role in the pathophysiologic process of neuropathic pain and chronic itch. Indeed, researchers have currently made significant progress in knowing the role of glial cells and the pro-inflammatory mediators produced from glial cells in the modulation of chronic pain and itch signal processing. Here, we provide an overview of the current understanding of neuroinflammation in contributing to the sensitization of the peripheral nervous system (PNS) and central nervous system (CNS). In addition, we also summarize the inflammation mechanisms that contribute to the pathogenesis of diabetic itch, including activation of glial cells, oxidative stress, and pro-inflammatory factors. Targeting excessive neuroinflammation may provide potential and effective therapies for the treatment of chronic neuropathic pain and itch in DM.

## Introduction

Diabetes mellitus (DM), one of the most serious metabolic diseases, is becoming the largest global epidemic of the 21st century, which causes multiple serious complications, such as neuropathic pain and diabetic itch. DM seriously affects the lives and economics of individuals, families, and societies (Stratton et al., 2000; Madsen et al., 2019; Calcutt, 2020; Rayego-Mateos et al., 2020; Schmitz et al., 2021). Diabetic neuropathy is one of the most prevalent comorbidities in patients with type 1 diabetes mellitus (T1DM) and type 2 diabetes mellitus (T2DM), which results in chronic pain and itching (Dewanjee et al., 2018; Zakin et al., 2019). More than 50% of diabetic patients develop diabetic neuropathy (Papanas and Ziegler, 2015; Feldman et al., 2019). Diabetic peripheral neuropathy (DPN), as the common form of diabetic neuropathy, leads to neuropathic pain with a characteristic “stocking-glove” pattern. Neuropathic pain, one type of chronic pain, is caused by a lesion or dysfunction of the peripheral or central somatosensory nervous system (Calcutt, 2020). Over one-third of patients with diabetic neuropathy develop neuropathic pain (Veves et al., 2008; Bansal et al., 2014). In recent years, diabetic neuropathic pain (DNP) is getting more and more attention, numerous studies have been conducted to identify the underlying pathological mechanisms in the hope of developing related therapeutic targets, even if the procedure is full of challenges and failures.

Itching (also termed pruritus) is the irritating sensation in the skin that initiates a desire for scratching (Ikoma et al., 2006; Lee et al., 2016; Dong and Dong, 2018). Patients with systemic diseases such as skin, kidney, or liver diseases suffer from chronic itching that is debilitating and has a serious impact on their quality of life (Yosipovitch and Bernhard, 2013). Chronic itching is also a common symptom of diabetic neuropathy. Unfortunately, the etiology of itching involved in diabetic neuropathy remains poorly understood and therapeutic strategy is inadequate.

Hallmarks of neuroinflammation includes the infiltration of immune cells, as well as the activation of glial cells [e.g., Schwann cells (SCs), satellite glial cells (SGCs), microglia and astrocytes], and increased of inflammatory mediators (e.g., pro-inflammatory cytokines, chemokines) in the peripheral nervous system (PNS) and central nervous system (CNS). Accumulating evidence suggests that neuroinflammation plays a significant role in the pathogenesis and progression of chronic pain and itching (Ellis and Bennett, 2013; Ji et al., 2014; Perera et al., 2015; Borghi et al., 2019; Cevikbas and Lerner, 2020), particularly neuroinflammation-driven sensitization contributes to the development and maintenance of DNP and chronic itching.

Here we review the current progress of neuroinflammation in PNS and CNS that contributes to the induction and maintenance of DNP, as well as existing treatment therapies for this pain. We highlight the important roles of neuroinflammation-driven sensitization involved in DNP. In addition, the neuroinflammation mechanisms contributing to the pathogenesis of diabetic itching are also summarized. An expanding understanding of the contribution of neuroinflammation-driven neuropathic pain and chronic itching in diabetes is helping to identify new therapeutic targets for the treatment of neuropathic pain and chronic itch in diabetes.

## Definitions and Terms Associated With Diabetes-Induced Pain and Itch

To better understand this review, some definitions, and terms associated with diabetes-induced pain and itching are listed in Table 1.

**TABLE 1:** Terms and related definitions or description.

Terms	Definitions or description	References
Neuropathic pain	The pain caused by a somatosensory nerve lesion or disease	Haanpää et al. (2011)
		Loeser and Treede (2008)
Diabetic neuropathy	People with diabetes usually develop this neurodegenerative disorder that affects the sensory axons, autonomic axons, and some motor axons	Calcutt (2020)
Diabetic peripheral neuropathy (DPN)	The most common form of diabetic neuropathy is featured by injury to neurons, SCs, and blood vessels within the nerve. The consequence is distressing and costly clinical sequelae, such as leg amputations, foot ulcerations, and neuropathic pain with a characteristic “stocking-glove” pattern	Feldman et al. (2019)
Painful diabetic neuropathy (pDN)	Diabetics experience pain directly as a result of abnormalities in the somatosensory system	Tesfaye et al. (2013)
		Jensen et al. (2021)
Central sensitization	Increase in the sensitivity of neurons in the central pain or itch pathway to normal or subthreshold afferent input. When peripheral injury or inflammation occurs, persistent stimulation of nociceptors or pruriceptors leads to an increase in excitability of central pathways or a decrease in the activity of inhibitory pathways	Loeser and Treede (2008)
		Cevikbas and Lerner (2020)
Peripheral sensitization	The nociceptors and pruriceptors in the PNS have an increase in responsiveness or a decrease in threshold to the stimulation in their receptive fields	Loeser and Treede (2008)
		Gao and Ji (2010)
		Lavery et al. (2016)
Itch (pruritus)	An uncomfortable cutaneous sensation that initiates the desire to scratch	Ikoma et al. (2006)
		Lee et al. (2016)
Chronic itch	An unpleasant sensation that leads to intensive scratching lasting 6 weeks or longer	Cevikbas and Lerner (2020)

## Epidemiology

Approximately 6.9%–10% of the general population is affected by neuropathic pain (Bouhassira et al., 2008; van Hecke et al., 2014; St John Smith, 2018). The increasing incidence is probably due to the aging population, high incidence of diabetes, and improved survival of cancer patients with subsequent chemotherapy (St John Smith, 2018). There is a higher incidence of chronic neuropathic pain in female patients than in male patients (8% vs. 5.7%), and in adults over 50 years of age than in those under 49 years of age (8.9% vs. 5.6%) (Bouhassira et al., 2008). Diabetes Atlas (9th edition, United Nations, 2019) edited by the international diabetes federation (IDF) describes 460 million (prevalence is ∼9.3%) diabetic patients in the general population in 2019 (Saeedi et al., 2019), and more than half of these patients suffered from neuropathy (Dyck and Giannini, 1996; Pop-Busui et al., 2009; Callaghan et al., 2015), of whom ∼1/3 develop neuropathic pain (Daousi et al., 2004; Abbott et al., 2011; Bouhassira et al., 2013). The prevalence of painful diabetic neuropathy (pDN) is ranging from 10 to 50% of all DM patients, (Abbott et al., 2011; Bouhassira et al., 2013; Alleman et al., 2015; Truini et al., 2018), as shown in Table 2. The prevalence of pDN varies among different studies, many reasons are due to the differences, containing case definition criteria used, participants selected, sample size, and types of diabetes (Ziegler et al., 2014). Amazingly, a recent study has shown a higher prevalence of neuropathic pain in patients with pDN (73.11% of 1,547) in mainland China (Zhang et al., 2021), all these data demonstrate the seriousness of pDN in diabetic patients. Itching is also a relatively frequent symptom in patients with diabetes. Although itching has been first investigated in DM in the late 1920s, until now, literatures about diabetic chronic itching are still limited. An intense scratching habit lasting more than 6 weeks is classified as chronic itching (Cevikbas and Lerner, 2020). The prevalence of chronic itching in the general population is ∼22% (Weisshaar, 2016), and the prevalence in DM is quite various, ranging from 18.4 to 35.8% (Neilly et al., 1986; Ko et al., 2013; Stefaniak et al., 2021a; Stefaniak et al., 2021b). This huge difference can be attributable to inconsistent definitions, varied tools for itch evaluation, age, gender, and diabetic populations with different diabetes types.

**TABLE 2:** Prevalence of pain and itch in diabetes in different areas, assessment methods in different studies.

Patients and area	Number of diabetic patients	Prevalence (%)	Methods	Reference
Pain				
Patients with diabetes in northwest England	n = 15,692	21	Questionnaire (NSS and NDS)	Abbott et al. (2011)
Patients with diabetes in France nationwide	n = 766	20.3	Questionnaire (DN4 and MNSI), monofilament test	Bouhassira et al. (2013)
Patients with diabetes in United Kingdom	n = 350	16.2	Questionnaire (VAS and McGill Pain) and examination	Daousi et al. (2004)
Patients with diabetes in Italy	n = 816	13	Clinical examination and diagnostic tests	Truini et al. (2018)
Patients with T2DM in Denmark	n = 5,114	10	Questionnaire (DN4 and MNSIq)	Gylfadottir et al. (2020)
Itch				
Patients with diabetes in the United Kingdom	n = 300	18.4	Interviewed and clinical examination	Neilly et al. (1986)
Diabetic outpatients in Japan	n = 2,656	26.3	Questionnaire	Yamaoka et al. (2010)
Patients with T2DM in Taiwan, China	n = 385	27.5	Questionnaire	Ko et al. (2013)
Children with T1DM in Poland	n = 100	22	NRS and Questionnaire (4IIQ)	Stefaniak et al. (2020)
Patients with T2DM in Poland	n = 109	35.8	NRS and Questionnaire (4IIQ)	Stefaniak et al. (2021b)

DN4, Diabetic Neuropathy 4; NDS, neuropathy disability score; NSS, neuropathy symptom score; MNSI, Michigan Neuropathy Screening Instrument; VAS, visual analog scale; 4IIQ, Four-item Itch Questionnaire; NRS, numerical rating scale.

## Neuroinflammatory Mechanisms Underlying Diabetes-Related Neuropathic Pain

Accumulating evidence suggests that neuroinflammation is closely related to chronic pain responding to stimuli (Perera et al., 2015; Ji et al., 2016; Ji et al., 2018; Borghi et al., 2019). The inflammation in PNS and CNS is characterized by the following: 1) an increase in the permeability of the blood-spinal cord barrier and blood-brain barrier (BBB), 2) infiltration of leukocytes, as the outcome of increased vascular permeability, 3) secretion and production of pro-inflammatory mediators (e.g., pro-inflammatory cytokines or chemokines), and 4) activation of glial cells causing the production of glial mediators that can regulate pain sensitivity (Ellis and Bennett, 2013; Ji et al., 2013; Ji et al., 2014).

It is well known that chronic pain results from neuronal plasticity in pain processing pathways. Neuronal plasticity involved in pain signal transmission consists of peripheral sensitization and central sensitization (Hucho and Levine, 2007; Basbaum et al., 2009; Gold and Gebhart, 2010; Woolf, 2011; Luo et al., 2014). Next, we highlight the important roles of neuroinflammation in promoting peripheral sensitization and central sensitization and involvement in DNP.

### Neuroinflammation and Peripheral Sensitization in DNP

As a result of inflammation and tissue injury, the critical characteristic of peripheral sensitization of nociceptors is presented by a decrease in threshold and an increase in response to noxious stimuli and spontaneous activity (Rosenberger et al., 2020). The hyperexcitability of sensory neurons in both patients and rodent models with diabetes presents as spontaneous activity and an altered stimulus-response function (Thrainsdottir et al., 2003; Kim et al., 2012; Nowicki et al., 2012). The presence of this aberrant activity is essential to the development and maintenance of DNP.

**FIGURE 1:**
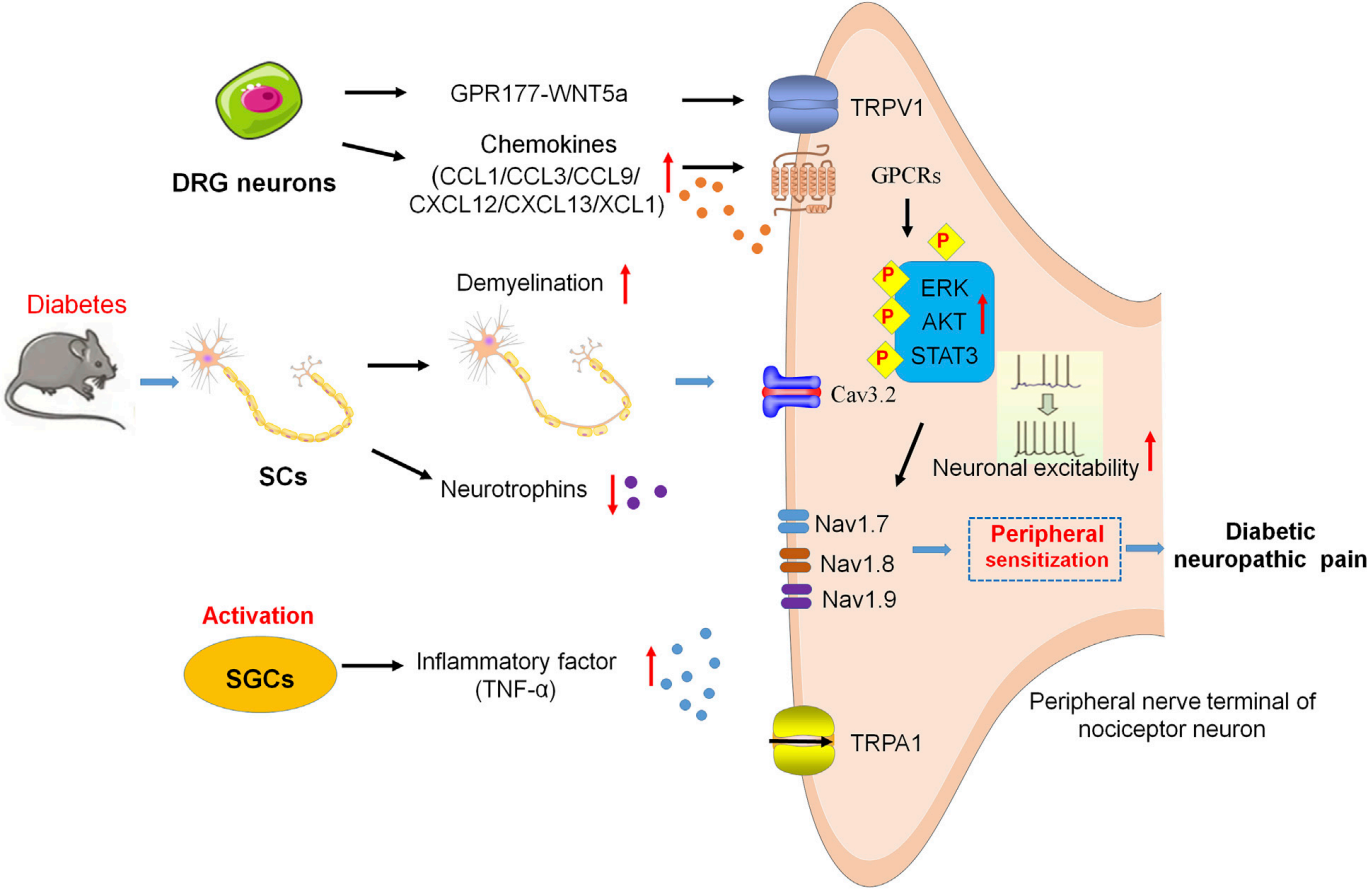
Neuroinflammation and peripheral sensitization in DNP. SCs are damaged and decrease neurotrophin expression, resulting in demyelination of axons and failure of nerve regeneration. SGCs release TNF-α and then enhance the excitability of peripheral nociceptive neurons. Cav3.2 activity results in hyperexcitability of DRG neurons via the glycosylation of extracellular arginine residues in diabetes. Chemokines activate the signaling cascades, such as ERK, AKT, and STAT3, to sensitize the Nav1.7, Nav1.8, Nav1.9, and TRPA1, leading to hypersensitivity and hyperexcitability of peripheral nociceptive neurons. In addition, GPR177 derives DNP via WNT5a/TRPV1 interaction.

Increasing reports suggest that SCs which ensheath the nerve fibers in the PNS are vital victims in the state of chronic hyperglycemia, causing demyelination in patients with diabetic neuropathy (Gumy et al., 2008; Dunnigan et al., 2013). SCs express both neurotrophins and their receptors. However, in diabetic patients or rodent models of diabetes, the robust decrease of neurotrophins in SCs results in unable to guide and support the regeneration of nerve fibers (Leinninger et al., 2004; Richner et al., 2014). In one previous study, streptozotocin (STZ)-induced diabetes reduces the level of ciliary neurotrophic factor (CNTF), an important neurotrophic factor from SCs (Calcutt et al., 1992). Some other studies have suggested that SCs and T cells interact with each other in diabetes. *Tang* et al., reported that levels of CXCR3 and phosphalated-p38 (p-p38) in the peripheral blood mononuclear cell (PBMC) of DPN patients are significantly increased. CXCR3 is elevated in CD8 (+) T cells via the p-p38 under high glucose conditions, and then promotes CD8 (+) T-cell recruitment into the diabetic nerves by CXCL9, CXCL10, and CXCL11 produced from glucose-stimulated SCs. Furthermore, results demonstrated that the upregulation of TNF-α, FasL, and PD-L1 in CD8 (+) T cells stimulating with SCs, which, in return, induce significant apoptosis of SCs, indicating the interaction of CD8^+^ T cells and SCs plays a key role in the development of DPN (Tang et al., 2013) (Figure 1).

Several previous studies have revealed that SGCs in ganglia are important for PNS functionality and glia activation. SGCs contact with each other and enwrap neuronal soma in ganglia. Deterioration of this communication among SGCs under pathological conditions leads to abnormal pain signal transmission (Dublin and Hanani, 2007; Huang et al., 2013). In T1DM and T2DM mice, the increased levels of glial fibrillary acidic protein (GFAP) are considered as the activation of SGCs, which have been shown to be associated with the induction of neuropathic pain (Hanani et al., 2014; Liu et al., 2016). In T2DM rats, the upregulation of purinergic signaling promotes the activation of SGCs, increases tumor necrosis factor-alpha (TNF-α) release from SGCs, and enhances the excitability of dorsal root ganglion (DRG) neurons, which brings about the pain sensitivity (Liu et al., 2016; Gonçalves et al., 2018) (Figure 1).

The activity and status of ion channels within sensory neurons largely determine the transmission and processing of pain signals (Bennett and Woods, 2014; Waxman and Zamponi, 2014). Ion channels [e.g., voltage-gated sodium channels (Nav), potassium channels, calcium channels (Cav), and transient receptor potential channels (TRP)] are participated in resting and action potentials (Waxman and Zamponi, 2014). In peripheral sensory neurons, three particularly prevalent Nav-isoforms are identified and named as Nav1.7, Nav1.8, and Nav1.9 (Dubin and Patapoutian, 2010; Hameed, 2019). In addition to setting the excitability of the terminal, Nav1.7 and Nav1.9 also function as threshold channels for amplifying the sensory signal, while Nav1.8 plays the role in the upstroke of action potentials in nociceptors (Blair and Bean, 2002). Potassium channels act as important breaks in the excitability of sensory neurons. T-type Ca2+ channels have also been found to play an important role in pDN by regulating the excitability of nociceptors in the subthreshold range. The activity of Cav3.2 is increased in diabetes via the glycosylation of arginine residues within extracellular membranes, which causes DRG neurons to be hyperexcitable. (Orestes et al., 2013). The changes in ion channels such as genetic variants, epigenetic modification, and abnormal expression, have all been implicated in the pathogenesis of neuropathic pain. *Sun et al.* reported that the increased expression of Nav1.8 is implicated in pDN, and such an increase reduces the failure probability of conduction in unmyelinated C fiber nociceptors, and then promotes more impulse conduction to the CNS, which results in neuropathic pain (Sun et al., 2012). Transient receptor potential vanilloid receptor-1 (TRPV1) ion channels are the important molecules involved in peripheral sensitization and pain modulation of chronic pain, which are widely expressed in nociceptive DRG neurons (Moore et al., 2018). A recent study reported that the orphan G protein-coupled receptor 177 (GPR177)-mediated wingless-related mammary tumor virus integration site 5a (WNT5a) secretion from A-fiber DRG neurons drives DNP by directly activating the TRPV1 channel and resulting in rapid currents and calcium elevations in DRG neurons (Xie et al., 2022). GPR177 and WNT5a are also found co-expressed in human DRG neurons, and pain intensity is positively related to WNT5a secretion in cerebrospinal fluid (CSF) among DNP patients (Xie et al., 2022). DNP is alleviated by interfering with WNT5a/TRPV1 interaction, thus providing a potential therapeutic target and intervention strategy for the clinical treatment of DNP (Xie et al., 2022) (Figure 1).

In addition, patients with diabetes have higher levels of reactive metabolites such as methylglyoxal (MGO), which post-translationally modify Nav1.8, then result in sensory neuron hyperexcitability, and finally lead to the development of diabetic pain (Bierhaus et al., 2012; Hansen et al., 2015). Rodent models of pDN showed signs of hypersensitivity in response to MGO via the activation of the sodium channel Nav1.8 and the transient receptor potential channel ankyrin 1 (TRPA1) (Bierhaus et al., 2012; Huang et al., 2016). It has been reported that MGO regulates the BBB permeability by producing the redistribution of junctional proteins, containing claudin-5 and β-catenin (Tóth et al., 2014), resulting in an increase in brain vessel permeability to MGO (Li et al., 2013). One previous research demonstrated that MGO specifically affects the integrated stress response (ISR) in IB4 positive DRG neurons *in vitro* and vivo diabetic models. The mechanical hypersensitivity of diabetic mice induced by MGO is attenuated by blocking the ISR (Barragán-Iglesias et al., 2019) (Figure 1).

### Neuroinflammation and Central Sensitization in DNP

Increasing studies also suggest that neuroinflammation-drives central sensitization play a crucial role in the neuropathic pain via acting on both PNS and CNS of diabetics (Loeser and Treede, 2008; Ji et al., 2018). The key features of neuroinflammation in CNS are the activation of glial cells (e.g., astrocytes and microglia), resulting in the upregulation of inflammatory mediators such as pro-inflammatory cytokines and chemokines. These chemokines and cytokines work as potent neuromodulators in the CNS that play a key role in triggering and maintaining the hyperalgesia and allodynia under chronic pain conditions (Samad et al., 2001; Kawasaki et al., 2008; Gao et al., 2009).

**FIGURE 2:**
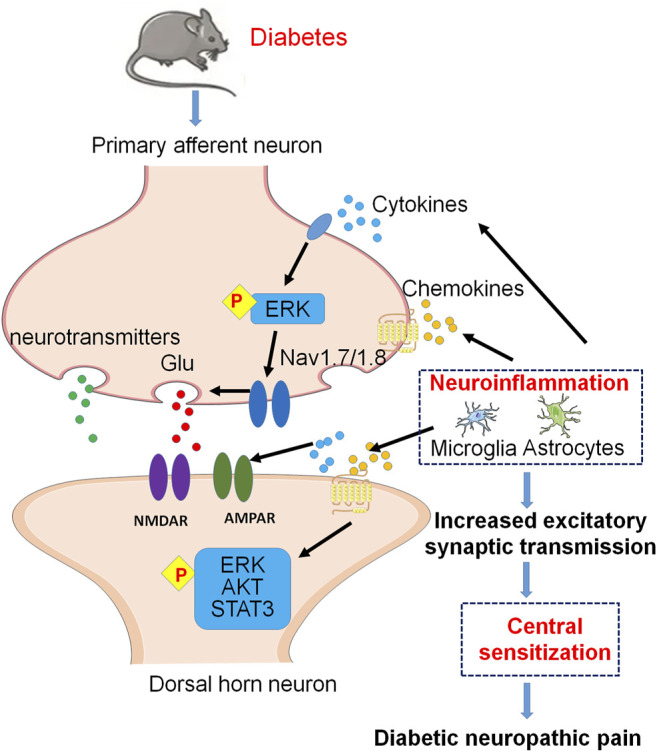
Molecular mechanisms of neuroinflammation and central sensitization in excitatory synapses of the spinal dorsal horn under DNP. Cytokines and chemokines from spinal glial cells activate pERK in primary afferent terminals and finally enhance glutamate (Glu) release via activation of Nav1.7 and Nav1.8. At postsynaptic membrane, activation of postsynaptic Glu receptors contributes to central sensitization. In addition, cytokines and chemokines activate postsynaptic pERK, pAKT, and pSTAT3 signaling pathways, which contribute to central sensitization of DNP.

In diabetic neuropathy, synaptic transmission within the spinal cord is increased by enhancing the input from spontaneously active nociceptors, which further amplifies nociceptive signaling (Woolf, 2011). It is also believed that this occurs because of a temporal and spatial accumulation of nociceptive signal inputs, causing the neurons in the spinal dorsal horn to have a heightened response to their inputs. Under diabetic neuropathy conditions, microglial cells transform to a pro-inflammatory phenotype, which releases pro-inflammatory factors [e.g., TNF-α, interleukin (IL)-6, IL-1β] and brain-derived neurotrophic factor (BDNF), further amplify nociceptive signal transmission in the spinal dorsal horn, and promote mechanical hypersensitivity in pDN (Tsuda et al., 2008; Salter and Beggs, 2014; Sun et al., 2015; Liu M et al., 2019). Consistent with the microglia activation, the activation of astrocytes is also enhanced in diabetic mice (Liu M et al., 2019). In the T2DM animal model, there is a correlation between ERK activation [phosphorylated ERK (pERK)] in spinal superficial neurons and astrocytes and hypersensitivity to pain, and pERK inhibition may provide a new treatment for diabetes-related pain (Xu et al., 2014). In addition, peripheral inflammation accompanied by prolonged nociceptive stimulation also increases the release of neurotransmitters [e.g., glutamate, BDNF, calcitonin gene-related peptide, and substance P] from the peripheral sensory fibers into the spinal dorsal horn and trigeminal nucleus. The increase of these neurotransmitters leads to the hyperexcitability of neurons in the spinal cord and supraspinal centers commonly referred to as central sensitization (Woolf, 1983; Woolf and Salter, 2000) (Figure 2).

An essential step for central sensitization is the activation of NMDARs and AMPARs at postsynaptic membrane surfaces (Ji et al., 2003; Latremoliere and Woolf, 2009). The previous study has shown that spinal activated astrocytes dramatically increase expression of IL-1β which may induce NMDAR phosphorylation in spinal dorsal horn neurons to enhance pain signal conduction in *db/db* mouse used widely as an animal model of T2DM. Therefore, the Astrocyte-IL-1β-NMDAR-Neuron axis unveils a novel mechanism underlying astrocyte-induced allodynia (Liao et al., 2011).

### Chemokines and Chemokine Receptors Involved in Diabetic Neuropathic Pain

Under both normal and pathological conditions, chemokines contribute to cell survival, proliferation, and inflammation via activating intracellular signaling pathways (Jiang et al., 2020). Accumulating evidence suggests that chemokines and their receptors also contribute to chronic pain via enhancing neuroinflammation in the PNS and CNS (Van Steenwinckel et al., 2011; Zhang et al., 2012; Zhang et al., 2013; Zhang et al., 2017; Fyfe, 2018; Lin King et al., 2019; Lu et al., 2021). Studies in the past decade have shown that several chemokines and their receptors are implicated in the pathogenesis of DPN, and associated signaling pathways of the chemokine pairs are involved in the mechanisms of diabetic neuropathy pain (Menichella et al., 2014; Jiang et al., 2016; Zychowska et al., 2017; Jayaraj et al., 2018; Rojewska et al., 2018; Liu S et al., 2019) (Figure 2).

Previous studies have demonstrated the crucial role of CCL1 in the pathogenesis of diabetic neuropathy caused by STZ. As a mediator of neuroimmune interactions, CCL1 plays an important role in the DNP through CCL1/CCR8 cross-talk (Zychowska et al., 2017). In a study of STZ-induced diabetes mice, CCL3 and CCL9 levels are increased in the lumbar spinal cord, while neutralizing antibodies against CCL3 or CCL9 delay neuropathic pain symptoms following STZ administration, and the application of CCR1 antagonist also alleviates pain-related behavior in diabetic neuropathy (Rojewska et al., 2018).

In the high-fat diet (HFD)-induced mouse model of T2DM, the increase of CXCL12 expression is detected in DRG neurons, and CXCL12/CXCR4 signaling contributes to the development of pain in diabetes through enhancing calcium influx and excitability of Nav1.8 positive DRG neurons, as well as promoting inflammatory cell infiltration (Menichella et al., 2014). Reducing CXCR4-mediated nociceptor hyperexcitability can reverse pDN in HFD mice, suggesting that CXCR4 in Nav1.8 positive DRG neurons is involved in the development of mechanical allodynia in HFD-induced diabetes (Jayaraj et al., 2018).

Our data have shown the spinal CXCL13/CXCR5 axis participates in neuropathic pain. Through neuron-to-astrocyte cross-talk, CXCL13 is upregulated in spinal neurons after spinal nerve ligation and activates spinal astrocytes by interacting with its receptor CXCR5 (Jiang et al., 2016). In the spinal dorsal horn of *db/db* mice with thermal hyperalgesia and mechanical allodynia, the CXCL13 and CXCR5 are also significantly increased, and the phosphorylation of cell signaling kinases, including pERK, phosphorylated AKT (pAKT) and phosphorylated signal transducer and activator of transcription proteins 3 (pSTAT3) are upregulated. Further evidence showed that CXCL13/CXCR5 signaling contributes to diabetic pain *via* activating pERK, pAKT, and pSTAT3 cell signaling pathways and promoting the production of TNF-α and IL-6 in the spinal cord of diabetic mice (Liu S et al., 2019).

The expression of XCL1 and XCR1 in the lumbar spinal segments (L4 to L6) of the STZ-induced DPN mice is increased. More evidence suggested that XCR1 is expressed mainly on neurons in the pathology of DN. XCL1 intrathecal injection enhances nociceptive transmission in naive mice, and XCL1 neutralizing antibody administration diminishes allodynia/hyperalgesia in STZ-induced diabetic mice (Zychowska et al., 2016).

### Advanced Glycation End-Products Involved in DNP

High levels of glucose lead to the glycation of several functional and structural proteins, resulting in producing advanced glycation end-products (AGEs). AGEs change gene expression and activation of nuclear factor-κB (NF-κB) via interacting with AGE-specific receptor (RAGE), thus inducing pro-inflammatory cytokines (e.g., IL-1α, IL-6, and TNF-α) (Neumann et al., 1999; Singh et al., 2014). In the spinal dorsal horn, TNF-α and IL-1β can act as neuromodulators to induce spinal synaptic plasticity such as long-term potentiation, and further promote neuropathic pain (Sorge et al., 2015; Taves et al., 2016).

## Neuroinflammatory Mechanisms Underlying Diabetes-Related Chronic Itch

Itch is an unpleasant cutaneous sensation that is accompanied by scratching or the desire to scratch (Ikoma et al., 2006; Lee et al., 2016; Dong and Dong, 2018). However, many similarities have been found between chronic pain and chronic itch (Ji, 2015; Moore et al., 2018; Ji et al., 2019). The cell bodies of itch sensory neurons are also located in the DRGs and trigeminal ganglia, and most itch neurons belong to C-type neurons (Ringkamp et al., 2011; LaMotte et al., 2014). The itch signals are generated in the primary afferent sensory fibers in the skin and then transmitted through the DRG neurons to the spinal dorsal horn neurons, and finally to the brain neurons (Ikoma et al., 2006; Han and Dong, 2014). Over the past decade, extensive research has been conducted on the mechanisms of itch, including peripheral and central neural mechanisms such as receptors and pathways involved in itch perception (Dong and Dong, 2018). According to the researchers, there are two main causes of the itch in diabetics, containing skin xerosis and diabetic polyneuropathy, suggesting that itch originates from dermatology or neurology (Stefaniak et al., 2021b). Additionally, oxidative stress and nerve inflammation contribute to diabetic polyneuropathy (Hagen and Ousman, 2021).

**FIGURE 3:**
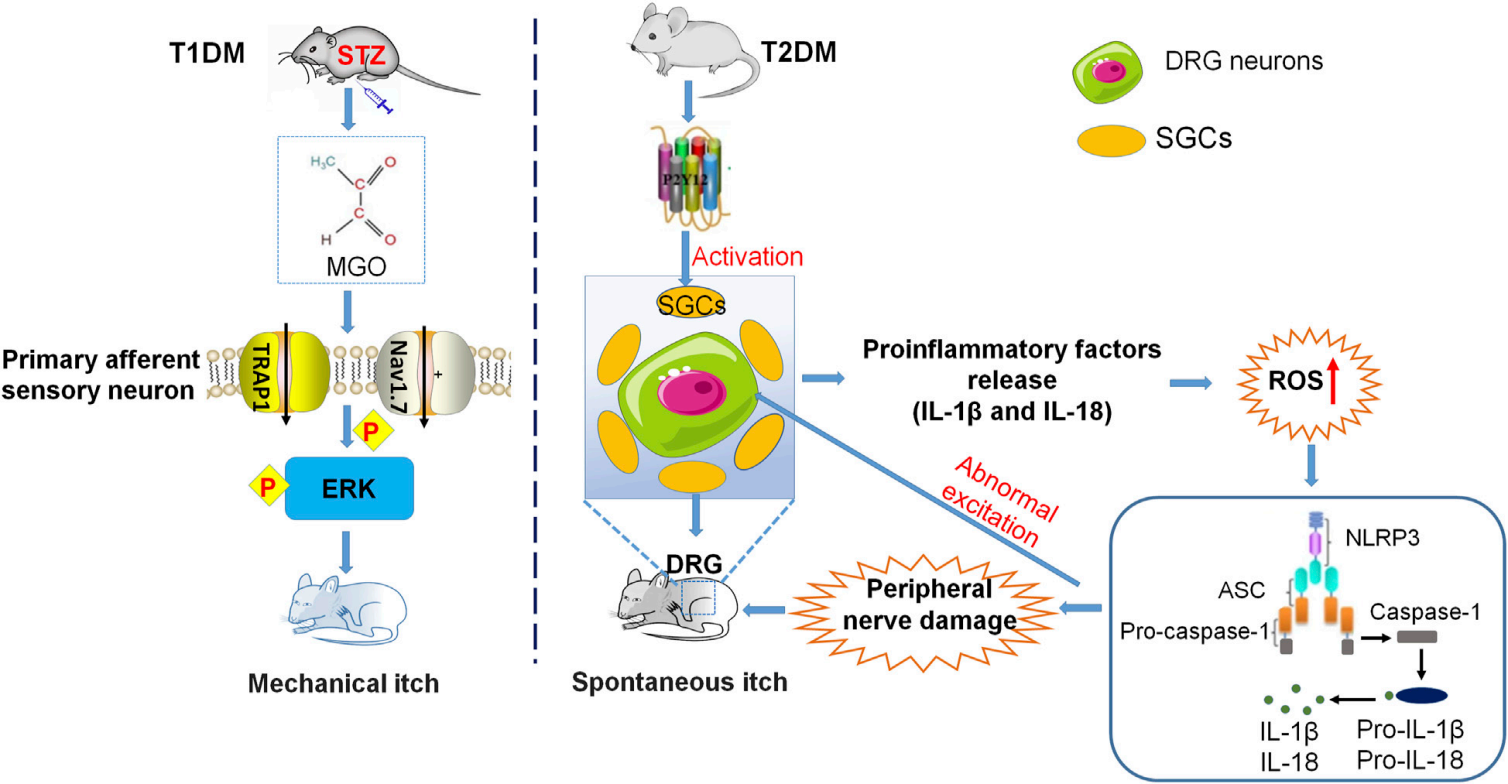
Neuroinflammatory mechanisms underlying diabetes-related chronic itch. The mechanical itch induced by MGO or in the STZ-induced mouse model of T1DM is mediated by activation of TRPA1, Nav1.7, and ERK in the DRG neurons. In the T2DM mouse model, the upregulation of P2Y12 expression in SGCs contributes to the increase of ROS, followed by the activation of NLRP3 inflammasome, the upregulation of inflammatory cytokines, and the damage to peripheral nerves. These changes finally result in DRG neuron hyperexcitability and sensitization.

Previous data have shown that sensitization is also a common mechanism in itch processing. Peripheral sensitization caused by the C fibers in the epidermis plays important role in pruritus sensitization (Ikoma et al., 2006; Tominaga and Takamori, 2014). Several other results indicated that spinal sensitization occurs frequently in atopic dermatitis (AD) model mice, but may not in psoriasis model mice (Shiratori-Hayashi and Tsuda, 2021). Mechanical itch (also known as touch-evoked itch) is a notable feature of chronic itch, and also a prominent mark in diabetic neuropathy (Bourane et al., 2015). Other evidence suggests that mechanical itch is related to central sensitization (Pan et al., 2019; Sakai and Akiyama, 2020). However, mechanisms of chronic itch in diabetes are not fully understood owing to inadequate related studies. Here, we summarize the inflammation mechanisms that participated in diabetic itch, including activity and status of ion channels, oxidative stress, and pro-inflammatory factors (Figure 3).

### Ion Channels Mediate Mechanical Itch in Diabetic Itch

Increasing evidence has suggested that TRPV1 and TRPA1 are the downstream effectors of itch‐related inflammatory factors and are involved in itch signals on the nerve fibers. During a pathological state, pruritus-related inflammatory factors such as IL-31, IL-4, and NGF, stimulate TRPV1 and TRPA1 repeatedly, resulting in a decrease in the threshold of itch sensation and causing chronic itch (Moore et al., 2018; Xie and Li, 2019). Both pain and itch are direct effects of immune dysfunction, since the release of pro-inflammatory mediators by immune cells and epithelial cells after tissue injury can directly activate or sensitize pain and pruritus neurons, causing hypersensitivity to pain and pruritus (Ji, 2015). Chronic itch and chronic pain caused by peripheral sensitization have been reported to be induced by inflammatory mediators, which require the activation of TRPA1 and Nav1.7 (Basbaum et al., 2009). It is widely recognized that MGO is a potential mediator of itch in diabetes. Incubation of MGO induces inward currents and calcium influx in TRPA1-expressing HEK293 cells or DRG neurons. (Cheng et al., 2019). Mechanical itch evoked by MGO or in STZ-induced T1DM mice is dependent on the activation of TRPA1, Nav1.7, and the pERK signaling pathway in DRGs and spinal cord (Cheng et al., 2019).

### Oxidative Stress Contributes to Diabetic Itch

Oxidative stress is an important factor in the pathogenesis of DM, especially in T2DM, which activates JNK, NF-κB, and p38 MAPK pathways to cause inflammation (Lamb and Goldstein, 2008; Agrawal and Kant, 2014). Previous studies have also shown that chronic and acute itching is related to oxidative stress (Liu and Ji, 2013; Zhou et al., 2019). ND7-23 cells (a cell line derived from the dorsal root ganglia) exhibit a significant increase in intracellular reactive oxygen species (ROS) after MGO treatment. MGO or STZ-induced mechanical itching is significantly reduced by intraperitoneal injection of antioxidant α-lipoic acid (ALA), indicating that oxidative stress contributes to diabetic itch (Cheng et al., 2019). Moreover, T2DM mice with chronic itch exhibit significantly higher levels of ROS in the DRG cells, suggesting that these compounds play an important role in diabetic itch (Xu et al., 2022).

### Pro-Inflammatory Factors in Diabetic Itch

Just like in chronic pain, pro-inflammatory factors such as cytokines and chemokines are also crucial in the pathogenesis of chronic itch (Liu et al., 2012; Storan et al., 2015). Diabetes was complicated by peripheral nerve injury results in an increase in the secretion of neuroinflammatory factors that can activate sensory C fibers and is accompanied by paraesthesia, suggesting that diabetic itch is due to abnormal discharges from damaged peripheral C fibers (Yamaoka et al., 2010; Yosipovitch and Bernhard, 2013). Spontaneous itching is an important indicator for evaluating itch behaviors. Recent studies have indicated that the number of spontaneous scratches in T2DM model mice is significantly increased. The increase of P2Y12 expression and SGC activity in these diabetic mice promotes the upregulation of ROS content, further activates the NLRP3 inflammatory body, and then produces inflammatory cytokines such as IL-18 and IL-1β. These inflammatory cytokines, in turn, cause peripheral nerve injury, abnormally excite DRG neurons, and result in spontaneous scratching. Treatment of P2Y12 shRNA or antagonist ticagrelor inhibits the spontaneous itch behaviors in the mouse model of T2DM (Xu et al., 2022).

## Strategies of Treatment

### Approaches to Treatment of Diabetic Pain

In recent years, targeted treatment of neuropathic pain is disappointing for a series of reasons as follows: 1) the underlying pathogenic mechanisms involved in neuropathic pain in diabetes are complex and not fully clarified, resulting in inadequate engagement of the claimed drug targets (Ji et al., 2014), 2) a translational gap from animal models of diabetes to patients with diabetes (King et al., 2009; Mogil, 2009), and 3) the serious side effects of existing analgesic drugs such as sedation, respiratory inhibition, tolerance, addiction and hyperalgesia following acute or chronic treatment.

Up to now, only glycemic control can prevent or slow down diabetic neuropathy progression in T1DM, but not in T2DM (Callaghan et al., 2012). Current evidence shows an association between diabetes and secondary complications with chronic inflammation. In addition to anti-inflammatory drugs, a multitude of hypoglycemic drugs such as thiazolidinediones, dipeptidyl peptidase-4 inhibitors, and metformin, have been found to reduce inflammation and improve outcomes. However, for all these hypoglycemic agents, it is necessary to distinguish between the anti-inflammatory effects produced by better glucose control and those related to the intrinsic anti-inflammatory effects of pharmacological compounds (Kothari et al., 2016).

According to the consensus from multiple guidelines and systematic reviews (Attal et al., 2010; Bril et al., 2011; Griebeler et al., 2014; Finnerup et al., 2015; Waldfogel et al., 2017), several drugs are supported to apply in the treatment of DNP, including calcium channel a2δ ligands (e.g., gabapentin and pregabalin) (Freeman et al., 2008; Moore et al., 2009; Griebeler et al., 2014; Finnerup et al., 2015; Pop-Busui et al., 2017), serotonin and noradrenaline reuptake inhibitors (SNRIs, e.g., duloxetine, venlafaxine) (Rowbotham et al., 2004; Wernicke et al., 2006; Zilliox and Russell, 2010; Tesfaye et al., 2013; Pop-Busui et al., 2017), and tricyclic antidepressants (TCAs, e.g., amitriptyline, nortriptyline, and desipramine) (Max et al., 1987; Max et al., 1991; Max et al., 1992; Boyle et al., 2012). However, these drugs do not clarify the potential pathogenesis for DNP.

Given the important roles of neuroinflammation such as cytokines and chemokines in the pathogenesis of DNP, targeting the pro-inflammatory mediators may provide a novel approach to treating DNP. There are three possible approaches for developing drugs that target chemokines and their receptors, including 1) blocking or neutralizing antibodies, 2) small-molecule inhibitors, and 3) small interfering RNA (siRNA). For example, antibodies that neutralize CCL3, CCL9, or XCL1 delay diabetic neuropathic pain symptoms. Similarly, CCR1 antagonist J113863 also attenuates pain-related behaviors in the diabetic pain model (Zychowska et al., 2016; Rojewska et al., 2018). Mechanical allodynia is alleviated in db/db mice following the injection of CXCR5 shRNA (Liu S et al., 2019).

More and more evidence has suggested that there is an inflammatory environment in the islets of patients with T2DM, including high levels of cytokines and chemokines, and immune cell infiltration. Therefore, many drugs targeting inflammatory cytokines such as TNF-α, IL-6, and IL-1β, are used to reduce insulin resistance and improve insulin secretion, further alleviating the complications of diabetes (Agrawal and Kant, 2014; Esser et al., 2015). For example, both troglitazone and gliclazide can reduce the TNF-α level in rodent models of diabetes. N-acetylcysteine (an anti-oxidant) attenuates the TNF-α levels in a dose-dependent manner, contributing to a decrease in the incidence and severity of diabetic neuropathy (Sagara et al., 1996). Tocilizumab (a monoclonal antibody targeting IL-6), drugs targeting IL-1β (e.g., anakinra, canakinumab, and other monoclonal antibodies), appear to reduce insulin resistance by reducing their pro-inflammatory effects in adipose tissue and muscle (Goldfine and Shoelson, 2017). Piroxicam statistically decreases the action potential amplitude of sensory neurons enhanced by STZ (Parry and Kozu, 1990). Nonsteroidal anti-inflammatory drugs (NSAIDs) reduce inflammation by inhibiting cyclooxygenase (COX) enzymes and are widely used in the prevention and treatment of T2DM (Bellucci et al., 2017). Moreover, drugs that target vascular endothelial growth factors (such as Pegaptanib and Avastin) and chemokines are used for the treatment of diabetic retinopathy (Kastelan et al., 2013). The current research studies shown that these drugs against pro-inflammatory mediators have certain therapeutic effects on diabetes, but cannot reverse the development of diabetes, overall, more studies are needed to validate these results. In addition, the selective blocking of Nav1.7 function has been successfully applied to trigeminal neuralgia, but the expected effect in diabetic neuropathy needs further to explore (Zakrzewska et al., 2017). The decrease of calcium influx via interfering Cav3.2 expression can also reduce pain hypersensitivity in diabetic mice (Messinger et al., 2009).

### Approaches to Treatment of Diabetic Itch

Currently, the mechanism involved in chronic itching, especially diabetic itching are poorly understood, resulting in limited effective therapies for chronic itching. Generally, treatment should be based on the therapeutic principle: finding out the cause, treating the primary diseases, avoiding the inducing factors, and moisturizing the skin (Greaves, 2005; Song et al., 2018). For diabetic itch, the optimal strategy is the treatment or prevention of causal diseases, that is, the maintenance of normal blood glucose (Steinhoff et al., 2018). In addition, some anti-inflammatory drugs targeting cytokines and chemokines (described in **6.1**) to treat the primary disease of diabetes probably also be beneficial to the treatment of itching induced by diabetes. Furthermore, experiments are needed in the future to confirm the anti-pruritic effect of these drugs on diabetes. In animal models of diabetes, knocking out of *Trpa1*, the blocker of Nav1.7, and TRPA1, antioxidants, and ERK inhibitor U0126 alleviate itching in mice evoked by STZ or MGO (Cheng et al., 2019). In addition, P2Y12 may be a promising target for the treatment of itching in T2DM (Xu et al., 2022).

Overall, drugs targeting diabetic itch patients are still inadequate, and further studies are needed to provide more information on the treatment efficacy.

## Perspective

As the most common chronic complication of DM, diabetic neuropathy results in chronic pain and itching. Our understanding of diabetic neuropathy continues to advance, especially neuroinflammation and sensitization-driven pain in diabetic neuropathy. However, the mechanism underlying pDN and chronic itching is still not fully revealed, hindering the development of therapies to treat diabetic pain and itch. Notably, chronic pain and itching are typically accompanied by anxiety, depression, and sleep disturbances, therefore, the development of drugs targeting inflammation not only helps treat diabetic pain and itching but also helps alleviate the development of mental illness in diabetic patients. Currently, many promising drugs in animal models or preclinical studies are aborted in clinical trials, which may be related to the insufficient representativeness of animal models, poor drug design, and design defects of clinical trials (Malik, 2016). Although regulatory agencies have approved a number of drugs and therapies to relieve the chronic pain and itch, it is worth noting that none of them are designed to target diabetes-specific mechanisms, while their efficacy varies from patient to patient and is confined to small subgroups of patients (Finnerup et al., 2010). Therefore, it is urgent and necessary to develop targeted drugs for diabetic pain and itching in the future.

## Abbreviations

AGEs, advanced glycation end-products; AMPA, α-amino-3-hydroxy-5-methyl-4-isoxazolepropionic acid; CNS, central nervous system; DM, diabetes mellitus; DPN, diabetic peripheral neuropathy; IL, interleukin; MGO, methylglyoxal; Nav, voltage-gated sodium; NMDA, N-methyl-D-aspartic acid; pDN, painful diabetic neuropathy; PNS, peripheral nervous system; SCs, schwann cells; SGCs, satellite glial cells; STZ, streptozotocin; T1DM, type 1 diabetes mellitus; T2DM, type 2 diabetes mellitus; TNF-α, tumor necrosis factor-alpha; TRPA1, transient receptor potential channel ankyrin 1.
